# Supplementary material for: Rab18 Drift in Lipid Droplet and Endoplasmic Reticulum Interactions of Adipocytes under Obesogenic Conditions
Source: Int J Mol Sci. 2023 Dec 6;24(24):17177. doi: 10.3390/ijms242417177 (PMC10743551; doi:10.3390/ijms242417177)
Supplement: Supplementary file 1 [file ijms-24-17177-s001.zip › Lopez-Alcala, Figure S1.pdf]

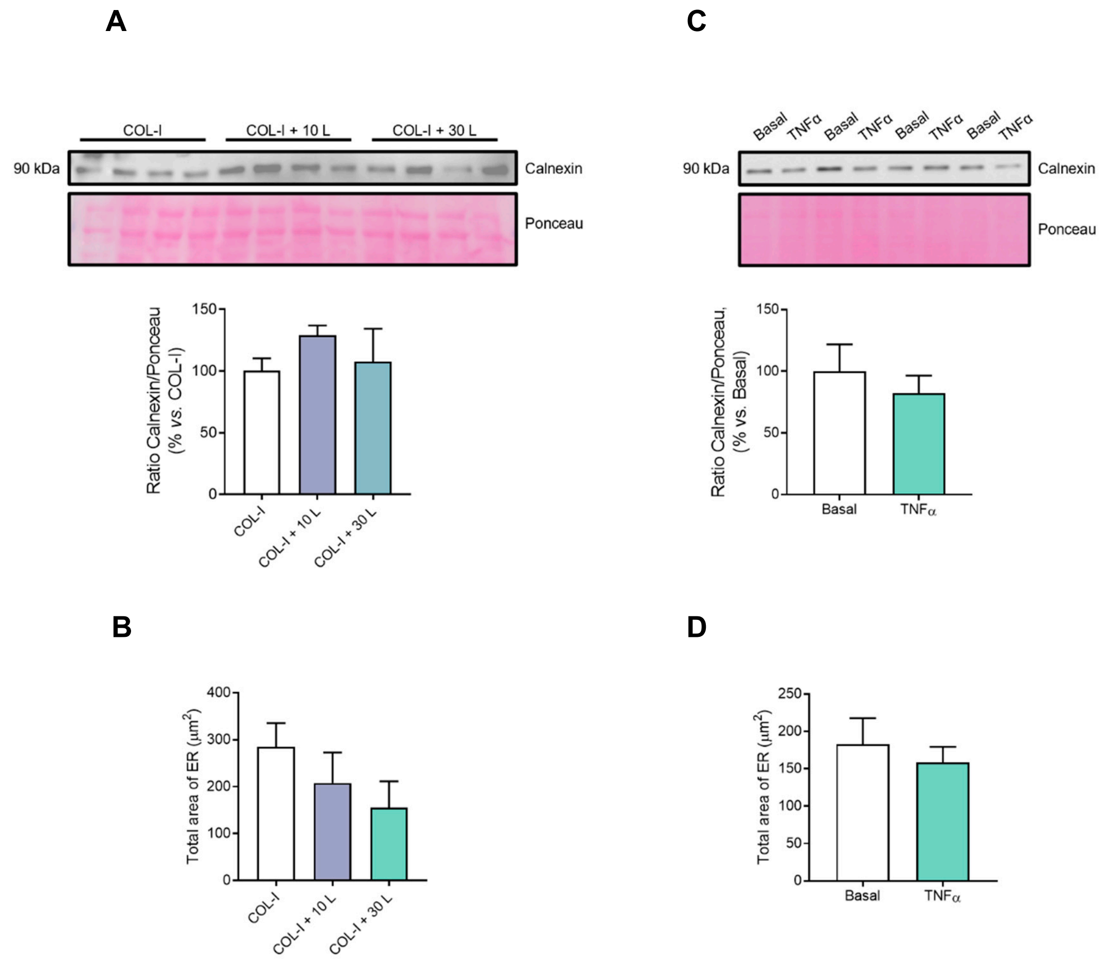

Figure S1. Analysis of the total mass of the endoplasmic reticulum in adipocytes under conditions of fibrosis and inflammation. (**A**, **C**) Representative immunoblots and their quantifications of calnexin (ER-marker) protein content in 3T3-L1 cells at D8 of differentiation under conditions of fibrosis (**A**) or inflammation (**C**). Ponceau staining was used as loading control (n=4-5 per condition). (**B**, **D**) Total area of ER per cell in 3T3-L1 cells under conditions of fibrosis (**B**) or inflammation (**D**) (n≥10 cells per condition from 2 independent experiments). Graphs show the mean ± SEM. (**A**) One-way ANOVA and Tukey's tests. (**B**) Kruskal-Wallis and Dunn's tests. (**C**, **D**) Independent t-test.
