# Supplementary material for: Rab18 Drift in Lipid Droplet and Endoplasmic Reticulum Interactions of Adipocytes under Obesogenic Conditions
Source: Int J Mol Sci. 2023 Dec 6;24(24):17177. doi: 10.3390/ijms242417177 (PMC10743551; doi:10.3390/ijms242417177)
Supplement: Supplementary file 1 [file ijms-24-17177-s001.zip › Lopez-Alcala, Figure S2.pdf]

Original immunoblots

Figure 2

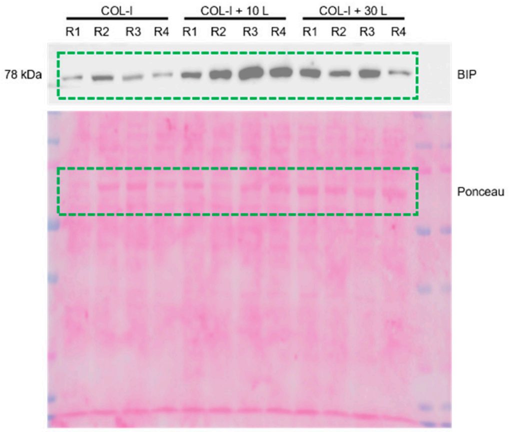

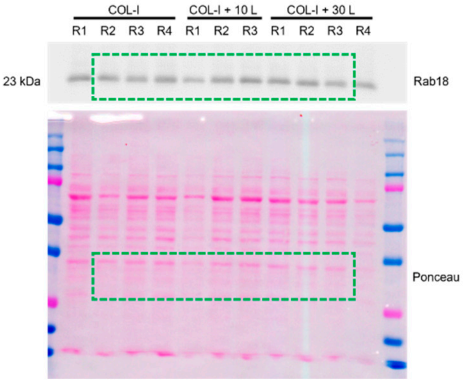

Figure 4

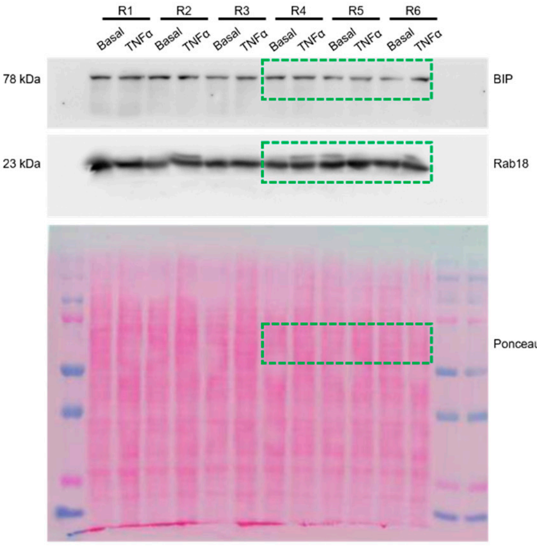

Figure S1

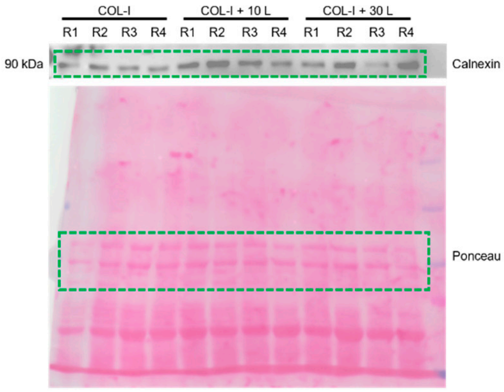

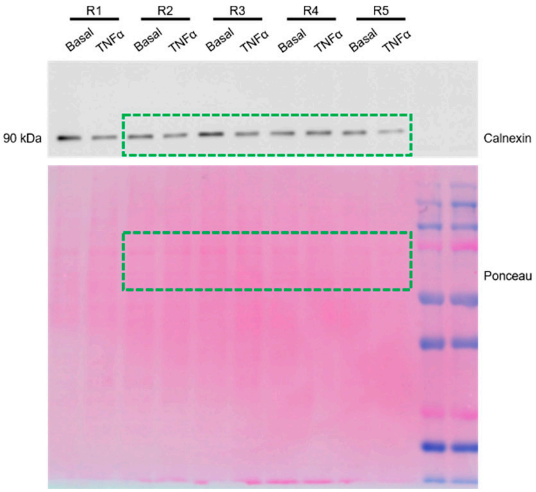

Figure S2. Uncropped scans of the immunoblots and Ponceau images from Figure 2, Figure 4 and Figure S1. The green dashed boxes indicate the regions shown in the corresponding figures.
