# Supplementary material for: Rab18 Drift in Lipid Droplet and Endoplasmic Reticulum Interactions of Adipocytes under Obesogenic Conditions
Source: Int J Mol Sci. 2023 Dec 6;24(24):17177. doi: 10.3390/ijms242417177 (PMC10743551; doi:10.3390/ijms242417177)
Supplement: Supplementary file 1 [file ijms-24-17177-s001.zip › Lopez-Alcala, Table S1.pdf]

Table S1. Clinical characteristics of the subjects with obesity included in the study.

n	7
Sex (male/female)	2/5
Age (years)	49 ± 3
Weight (kg)	129.59 ± 8.44
Height (m)	1.67 ± 0.05
BMI (kg/m^2)	46.10 ± 1.18
Body fat (%)	61.24 ± 2.60
Glucose (mg/dL)	110.14 ± 14.72
HbA1c (%)	5.63 ± 0.12
Triglycerides (mg/dL)	128.29 ± 24.06
Total cholesterol (mg/dL)	151.57 ± 10.06
LDL-cholesterol (mg/dL)	81.50 ± 7.53
HDL-cholesterol (mg/dL)	40.17 ± 1.92
CRP (mg/L)	9.61 ± 3.76
AST (IU/L)	19.83 ± 2.77
ALT (IU/L)	26.00 ± 5.65
Ferritin (ng/ml)	175.08 ± 42.09
Hematocrit (%)	40.54 ± 2.06
Haematids [million/mm^3]	4.62 ± 0.25
Platelets [million/mm^3]	0.25 ± 0.01

Data presented as mean ± SEM. AST, aspartate aminotransferase; ALT, alanine aminotransferase; CRP, high-sensitivity C-reactive protein.
